# Annotations

**Published:** 1910-10-01

**Authors:** 


### ANNOTATIONS.

### The Caravan Work of the "Aurora."

THE "Aurora," the first caravan of the Women's Imperial Health Association, which was inaugurated on August 20, has succeeded beyond the best hopes of the Executive Council. Nineteen towns and villages have been visited, and lectures and talks given in each, upwards of seven thousand people of the class which the lectures were designed to reach having been addressed. Large quantities of literature have been distributed, and it is a significant fact that none of it has been found lying about afterwards. Careful note is taken of the difference between the number of windows found closed in each town or village before the lectures, and the number found open afterwards; in every case there has been a marked increase. One notable and unique feature is that at Newbury a local lady offered two prizes to be awarded to children at Christmas who show the best care of their teeth. In all places visited the local authorities—Mayors, medical officers of health, and doctors—have evinced the greatest interest in the work of the caravan.

### A Question of Certificates.

A SOMEWHAT curious state of affairs recently arose at Mazamet, France, in consequence of an outbreak of anthrax among workers in the hide industry. No doubt can be entertained as to the existence of the disease, since the anthrax-bacillus was found in several samples of the exudates collected from the pustules of patients and sent to the Pasteur Institute for examination. The victims, refusing the ministrations of the regular practitioners of the town, put themselves under the care of a number of unqualified "healers," who, it is said, effected in some cases marvellous cures. As the result of this attitude, no certificates were forthcoming from qualified sources, and the insurance companies refused compensation for loss of wages, the employers, on the same grounds, declining to grant sick-pay. Since all parties insisted on sticking to the positions they had severally taken up on the matter, talks of a general strike were heard on all sides. In the meantime, however, a local irregular practitioner had offered to place his method of treatment at the disposition of the Pasteur Institute, only to find that his generosity did not meet with the enthusiastic reception he no doubt expected. As things were getting serious, the authorities had to take action, and a meeting of delegates of employers and employed was eventually arranged. After protracted discussion, in the course of which the legitimate susceptibilities of the medical faculty came in for some extremely impolite criticism, terms were arrived at and a strike avoided, at least for the time being. We must confess to being curious as to what would have happened in the event of these proceedings having proved abortive. Would the insurance companies have stuck to their guns and fought the cases in the Courts? Would they have won them? Or would the Judges have decided in favour of the workers, at the same time animadverting on the disgraceful obstinacy of medical men in refusing to recognise the advances of "science," as shown by the wonderful cures wrought by their unofficial "colleagues"?

### Milk Supply.

THE Secretary of the National League for Physical Education and Improvement has forwarded to these offices specimen copies of three leaflets designed with the object of improving the public health by ensuring the supply of clean milk. The sheets are addressed respectively to farmers and other milk producers, to distributors and retailers of milk, and to housewives and all consumers of milk. These are supplied at two shillings a hundred, or seventeen and six per thousand, and local authorities purchasing them for distribution can have their names inserted in a prominent position on the front page. The leaflets are packed full of most important facts and instructions, well displayed and emphasised by the use of leaded types in many places. They cover adequately, yet in simple language, the whole range of a very intricate subject, and one which is bound up most intimately with the future and present welfare of the race. We are not sanguine enough to believe that any considerable section of those to whom the leaflets are addressed will adopt fully the recommendations of the League, though we are amply convinced that all should do so, for even among well-educated people the carelessness and indifference which exist on this and kindred sanitary subjects can only be described as colossal. Yet since it is only by strenuous and continued effort that improvement will ensue, nothing but the heartiest approval and the best wishes for a successful propaganda can be extended to the League for their excellent efforts. It is especially among the children, whose ingrained prejudices against cleanliness and sanitation are less deep and more easily overcome, than with their parents and elders, that the best results will accrue. We would have every school child in the country instructed in leaflets A and C, and in town in leaflets B and C. Further, since the teachers are those on whose conviction and earnestness the success of all hygienic teaching depends, it would be well if every primary school-teacher could be supplied with copies of all three pamphlets, and compelled to master them. Lastly, we would have the medical profession keep hammering away at this question of milk supply perpetually, for though the laity often scoff at what they call grandmotherly methods, they can yet be convinced if they see their doctor is thoroughly and honestly in earnest. It is very often those who are most inclined to chaff their medical attendant over the "fads" of modern hygiene that really are most easily impressed and converted if they see him sticking to his text, and, above all, practising what he preaches. We would suggest that every doctor should begin by making it his own personal affair to see the simple rules laid down in pamphlet C of this series carried out in his own kitchen; and should then urge the same methods of milk preservation upon his patients.

